# Establishment of a digital PCR method for detection of *Borrelia burgdorferi* sensu lato complex DNA in cerebrospinal fluid

**DOI:** 10.1038/s41598-022-24041-8

**Published:** 2022-11-21

**Authors:** Trine Andreasen Leth, Sara Moeslund Joensen, Malene Bek-Thomsen, Jens Kjølseth Møller

**Affiliations:** 1 Department of Clinical Microbiology, Lillebaelt Hospital – University Hospital of Southern Denmark, Beriderbakken 4, 7100 Vejle, Denmark; 2 Department of Regional Health Research, Faculty of Health Sciences, University of Southern Denmark, Vejle, Denmark; 3 Department of Clinical Genetics, Aarhus University Hospital, Aarhus, Denmark

**Keywords:** Microbiology, Molecular biology, Diseases

## Abstract

Direct detection of *Borrelia burgdorferi* sensu lato bacteria in patient samples for diagnosis of Lyme neuroborreliosis (LNB) is hampered by low diagnostic sensitivity, due to few bacteria in cerebrospinal fluids (CSF) samples. Evaluation of novel molecular methods, including digital PCR (dPCR), as future tools in diagnostics of LNB is desirable. This study aimed to establish a dPCR assay and validate pre-PCR procedures for detection of *Borrelia* in CSF. Synthetic DNA fragments and cultured *Borrelia* reference strains were used during optimisation experiments. In addition, 59 CSF specimens from patients examined for LNB were included for clinical validation. The results showed that the pre-PCR parameters with the highest impact on *Borrelia*-specific dPCR method performance were incubation of the PCR-plate at 4 °C for stabilization of droplets, centrifugation for target concentration, quick-spin for dPCR rain reduction, and PCR inhibition by matrix components. *Borrelia* DNA in CSF was detected in one out of nine patients with LNB. Diagnostic sensitivity was determined to be 11.1% and specificity 100%. In conclusion, this study reports an optimized *Borrelia*-specific dPCR method for direct detection of *Borrelia* in CSF samples. The present study does not support the use of *Borrelia*-specific dPCR as a routine method for diagnosing LNB.

## Introduction

Lyme neuroborreliosis (LNB) is a nervous system disorder caused by bacteria belonging to the *Borrelia burgdorferi* sensu lato complex. The *Borrelia* bacteria (Bb) may disseminate from a tick bite and establish infection in the central nervous system (CNS) causing neurological disease manifestations^1,2^. The most common neurological manifestation of early LNB in Europe is lymphocytic meningoradiculitis, which is defined by painful radicular neuritis, cranial nerve abnormalities such as facial nerve palsy, and mononuclear cell CNS inflammation^3,4^. Various Bb can cause LNB including *B. burgdorferi* sensu stricto, *B. garinii*, *B. spielmanii*, *B. bavariensis*, and *B. afzelii*^5^.

Direct detection of Bb in cerebrospinal fluid (CSF) samples is challenging because the Bb are present in extremely low numbers and Bb culturing is laborious and may take up to 12 weeks^6^. The polymerase chain reaction (PCR) technique has, due to its turnaround time and high sensitivity and specificity, revolutionized the diagnostics of diseases with microbiological aetiology. However, studies using PCR in the diagnostics of LNB have shown variable results with clinical sensitivities ranging between 12 and 46%^7–10^. Hence, PCR is not recommended as a routine test for diagnosis of LNB^11^. Therefore, the diagnosis of LNB is based on neurological symptoms suggestive of LNB together with CSF pleocytosis and detection of intrathecally produced *Borrelia*-specific antibodies (antibody index, AI)^11^. The AI result can be negative in patients with a short duration of neurologic symptoms, due to limited synthesis of intrathecal antibodies^12^. In these early LNB cases, PCR-based technologies may serve as supplemental diagnostic tools, because the bacterial load in CSF is relatively higher early in the disease course^13^. To detect these early LNB cases by PCR, the analysis still needs to be highly sensitive and specific.

During the last decade, novel digital PCR (dPCR) platforms have become commercially available and affordable leading to rapid application in fields of clinical oncology, prenatal diagnostics, and infectious diseases^14^. The detection principle of the dPCR technique relies on target molecules that are randomly distributed across many small and independent partitions. In each partition with a target molecule, PCR is performed, resulting in partitions with high amounts of fluorescent signal that can be separated from the empty negative partitions with baseline signal only, thus resulting in a digital output. An evaluation of the dPCR technology as a future tool in diagnostics of LNB is desirable as dPCR has several advantages over conventional PCR, including the ability to detect DNA in samples with minimal target numbers using the concentration effect of the partitions and by enrichment of the target molecules from interfering compounds or background signals^15–17^.

Digital PCR assays for detection of Bb have been established in relation to ticks and blood samples^18,19^, but the dPCR technique has not yet been applied in the context of LNB.

In addition, suboptimal treatment of samples before the PCR analysis is performed (pre-analytical procedures) may have adverse effects on the PCR performance, and thus result in low diagnostic sensitivity^10,20^. Therefore, it is critical that the pre-analytical steps (concentration and purification) are evaluated and adjusted as well to optimize the entire PCR method. We hypothesise that a crude sample preparation in combination with a sensitive dPCR assay may be used for detection of Bb DNA in CSF specimens.

### AIM

The objective of this study was to establish an optimized dPCR method for Bb-specific detection in cerebrospinal fluid samples. This was done by systematically evaluating different analytical steps with a potential effect on overall dPCR method performance:

A. Establishment of *Borrelia* dPCR assay.
B. Optimization of “direct” pre-PCR method *without* DNA extraction.
C. Compare the “direct” method to the automated Maxwell DNA extraction method.
D. Test of patient samples with optimized pre-analytical method and dPCR assay.

## Results

### dPCR optimization workflow

**Figure 1:**
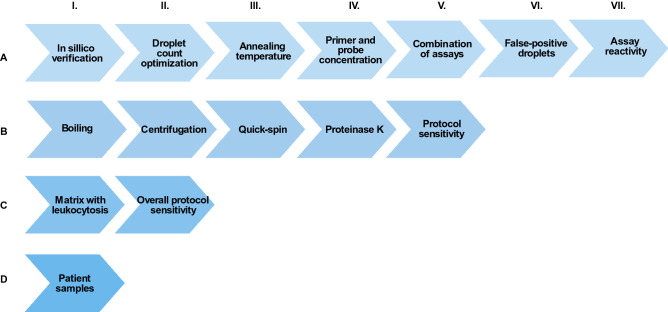
Workflow of the stepwise experimental setups during establishment of the *Borrelia*-specific digital PCR (dPCR) assay. The workflow highlights the different stages that must be performed to evaluate the robustness, specificity, and sensitivity of the *Borrelia* dPCR assay and pre-PCR method. The Roman numerals indicate the order of the experiments. (A) Establishment and validation of the in-house *Borrelia* dPCR assay. (B) Optimization of “direct” pre-PCR method without DNA purification. (C) Comparison of “direct” method to Maxwell DNA purification method. (D) Test of patient samples with optimized pre-analytical method and dPCR assay.

A detailed description of the experimental setups is presented in Supplementary. Our approach to establish and optimise a highly specific and sensitive *Borrelia* dPCR method are outlined in Fig. 1 and described below.

### *Borrelia* dPCR assay establishment and optimization (A)

For detection of the five *Borrelia* species (*B. burgdorferi* sensu stricto, *B. garinii*, *B. afzelii, B. spielmanii,* and *B. bavariensis*) known to cause LNB, we chose to use the *Borrelia*-specific primer and probe sequences described by Ornstein and Barbour in 2006. We performed an in-silico verification of the primers and probe in two stages. First, primers and probes were entered into the NCBI Nucleotide BLAST web application and checked against the nucleotide collection database (nr/nt) to look for sequence homology with different *Borrelia* strains as well as potential non-specific matching of particularly human or microbial origin. BLAST default settings were applied. Second, the primers and probes were checked for oligo-dimerization using the IDT OligoAnalyzer™ Tool (Integrated DNA Technologies Inc., USA). All primers and probes fulfilled our acceptance criteria, e.g., “Expect value”^21^ for sequence homology below 0.01.

During the initial dPCR experiments we observed that it was important to incubate the dPCR plates on the PCR thermal cycler at 4 °C, overnight, prior to reading to obtain a minimum of 15,000 droplets per well. When comparing the total droplet counts from plates that were read directly after PCR amplification (mean 13,519 droplets per well) to the total droplet counts from incubated plates (mean 17,524 droplets per well), the difference was statistically significant (p < 0.001).

**Figure 2:**
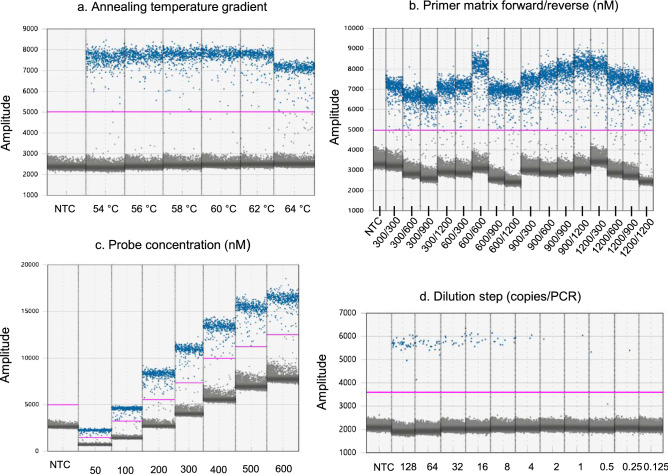
Examples of digital PCR (dPCR) output demonstrating the optimization of the *Borrelia burgdorferi* sensu lato dPCR assay. The same assay components and *Borrelia* gBlock template is used in all parts of the figure unless otherwise stated. Each column represents samples from individual wells and is separated by a grey line. Blue dots represent the positive partitions and grey dots represent the negative partitions. The horizontal pink lines illustrate the manual threshold setting for the data analysis. Amplitude on Y-axis refers to fluorescence intensity of individual partitions. NTC (non-template control) was molecular graded water in all experiments. All figures show one representing PCR reaction of each sample in the experiment unless stated otherwise. (**a**) The effect of the annealing temperature (gradient from 54 to 64 °C) on the amplification, and thus on the final amplitude of the positive and negative partitions. For each tested temperature, PCR samples containing NTC or approximately 600 copies of *Borrelia* gBlock were run in duplicates. (**b**) The effect of different concentrations of forward and reverse primers, on the difference in fluorescence amplitude between positive and negative partitions. The probe concentration was 200 nM in every PCR reaction. For each combination tested, PCR samples containing NTC or approximately 600 copies of *Borrelia* gBlock were run in triplicates. (**c**) The effect of different probe concentrations on the fluorescence intensity. PCR reactions contained 900 nM forward and 900 nM reverse primer. For each combination tested, PCR samples containing NTC or approximately 600 copies of *Borrelia* gBlock were run in triplicates. (**d**) Twofold dilution series for assessment of the analytical sensitivity. PCR reactions contained 900 nM forward and reverse primer and 300 nM probe. For each dilution step tested, PCR samples containing NTC or *Borrelia* gBlock were measured in eightfold.

The singleplex *Borrelia* dPCR assay was optimised using a stepwise approach to evaluate how the different parameters (Fig. 1A-III to A-V) affected the clustering (grouping of either positive or negative partitions), cluster separation (differentiation between positive and negative partitions), the amount of rain, and the quantification. Representative data from these experiments can be seen in Fig. 2a–c.

There was no difference when comparing temperatures between 54 and 62 °C (Fig. 2a). However, at 64 °C, the fluorescent amplitude difference between positive and negative partitions was markedly reduced. The optimal annealing temperature for the PCR amplification was determined to be 62 °C, to achieve the highest degree of target specificity as possible in combination with optimal cluster separation.

To determine the optimal concentrations of the forward and reverse primers, we tested different primer pair combinations of four forward primer concentrations (300–1200 nM) with four reverse primer concentrations (300–1200 nM) in a primer matrix. The resulting data (Fig. 2b) was extracted from the QuantaSoft software to Excel. The mean amplitude of the negative clusters was subtracted from the mean amplitude of the positive clusters to calculate the mean amplitude difference. The primer pair with the highest mean amplitude difference was 900 nM forward primer in combination with 900 nM reverse primer (Supplementary A, Table SA6).

The optimal probe concentration was determined using combinations of probe concentrations ranging from 50 to 600 nM, all tested in triplicates. Based on visual inspection of the results, 300 nM probe was determined to best separate the positive and negative partitions (Fig. 2c).

A fragment of the Phocid herpesvirus (PhHV) was used as target for the internal positive PCR control (IPC). First, the IPC assay was tested alone to assess the separation of the IPC positive cluster from the IPC negative cluster (data not shown). Then the *Borrelia* and the IPC assays were combined in a duplex to evaluate whether the assays affected each other. The evaluation was performed using an IPC concentration of 500 IPC copies per PCR reaction and high (500 copies), medium (50 copies) and low (5 copies) of *Borrelia* gBlocks per PCR reaction (Supplementary A Fig. SA1). We did not observe any difference regarding amplitude separation, rain or quantification of *Borrelia* gBlock targets, when comparing the *Borrelia*/IPC duplex assay to the *Borrelia* singleplex assay.

False-positive partitions are known to occur in dPCR experiments, due to underlying *Taq* errors. Therefore, we assessed the frequency of false-positive partitions by testing samples containing only PCR-grade water, the “negative CSF matrix”, or Maxwell elution buffer (Supplementary A, Fig. SA2). No false-positive partitions were detected in the samples with “negative CSF matrix” or Maxwell elution buffer, however in 5% (1/20) of the negative samples with PCR-grade water we observed one false-positive partition. Therefore, we determined that for all subsequent analysis, samples were to be run as duplicates and that a sample could only be classified as positive if (I) one of the duplicates had at least two positive partitions, or (II) if both duplicates had at least one positive partition each with the same fluorescence amplitude as the positive cluster of the positive control in the *Borrelia* assay.

The analytical reactivity describes the performance of the dPCR assay in terms of cross-reactivity and ability to detect the desired targets (specificity), together with an assessment of limit of detection (sensitivity). The analytical specificity was evaluated using a collection of purified bacterial and viral DNA from either laboratory culture strains or QCMD past panels, representing various important differential diagnostic CNS infections (Supplementary A, Table SA4) and a collection of *Borrelia* reference strains (Supplementary A, Table SA5). We did not detect any false-positives samples and the *Borrelia*-specific dPCR assay did detect all Bb reference strains (data not shown). The analytical sensitivity of the *Borrelia* assay was determined by a twofold dilution series using known copies of *Borrelia* gBlock in water (Fig. 2D). The limit of detection (LOD) was defined as the lowest number of *Borrelia* copies per PCR reaction, where at least two partitions were present in minimum four of the eight PCR replicates, or equivalent in at least eight positive partitions in the pooled eight replicates. The estimated analytical sensitivity was four copies of *Borrelia* gBlock in the PCR reaction in at least 15,000 total partitions. Lower amounts of *Borrelia* copies per PCR reaction were also detectable, but at a more infrequent rate.

### Optimization of “direct” pre-PCR method without DNA extraction (B)

**Figure 3:**
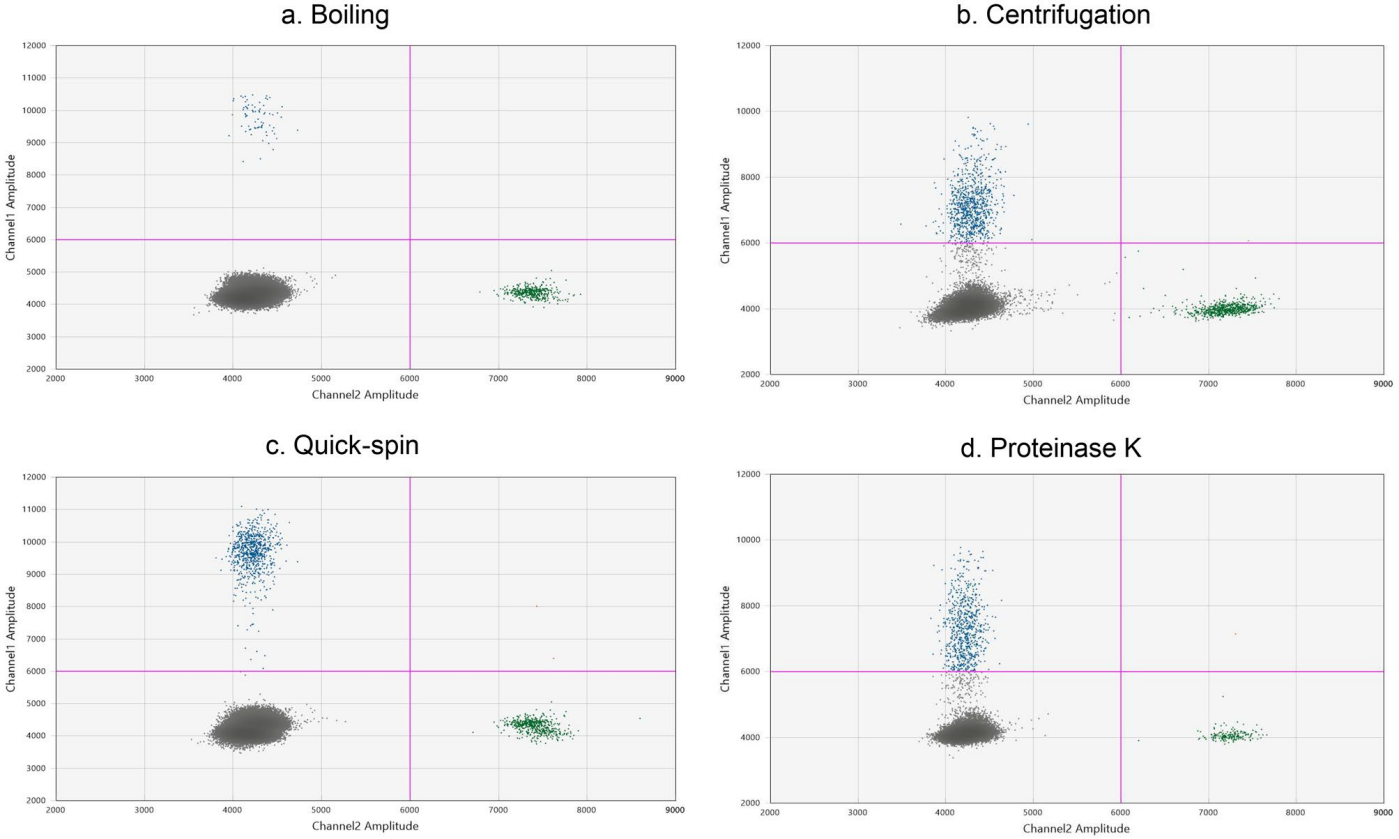
Optimization of a “direct” pre-PCR method for detection of *Borrelia* DNA in CSF matrix without purification presented in a 2D-plot. The blue and green dots represent the *Borrelia* positive partitions and IPC positive partitions respectively, and grey dots represent the negative partitions. The horizontal pink lines illustrate the manual threshold setting for the data analysis. Amplitude on X and Y-axis refers to fluorescence intensity of individual partitions. For every spike experiment *Borrelia garinii* strain Lu118 was counted and diluted with “negative CSF matrix”. Each figure shows the results of samples with a concentration of 100 Bb per mL. (**a**) *Borrelia* detection after the boiling lysis experiment. (**b**) The effect on *Borrelia* concentration and output when including a centrifugation step for concentration prior to boiling. (**c**) The effect on *Borrelia* rain, when including a quick-spin step in between sample boiling and dPCR reaction assembling. (**d**) The effect on fluorescence amplitude and rain, when subjecting the boiled sample to proteinase K treatment.

dPCR has been described to be less prone to PCR interference by matrix substances. Therefore, we optimized a method for direct detection of Bb spiked in negative CSF matrix. First, the spiked samples were boiled at 95 °C, cooled to 4 °C, and then the crude extracts were assessed by *Borrelia* dPCR as described in detail in Supplementary B. The assay was able to detect the released *Borrelia* DNA target (Fig. 3a) in concentrations down to 100 Bb per sample. A centrifugation step prior to boiling resulted in improved detection of Bb per sample (Fig. 3b) down to 10 Bb per sample, but it also induced more intermediate fluorescent partitions (termed rain). Both the improved sensitivity and the emerged rain were results of ten-fold concentration of the sample by the centrifugation step. To reduce the amount of rain occurring after the concentration step, we evaluated two approaches to minimize PCR inhibition, a quick-spin step, and a proteinase K treatment respectively. The quick-spin step effectively reduced the amount of rain and facilitated a good separation of the positive and negative clusters (Fig. 3c). The proteinase K treatment did not remove much of the intermediate partitions (Fig. 3d). Therefore, the quick-spin step was chosen as preferred method for rain reduction. Thus, the optimized direct method for detection of isolated *Borrelia* DNA without DNA extraction was as follows; centrifugation followed by boiling and cooling, and lastly a quick-spin prior to dPCR mixing and analysis.

**Figure 4:**
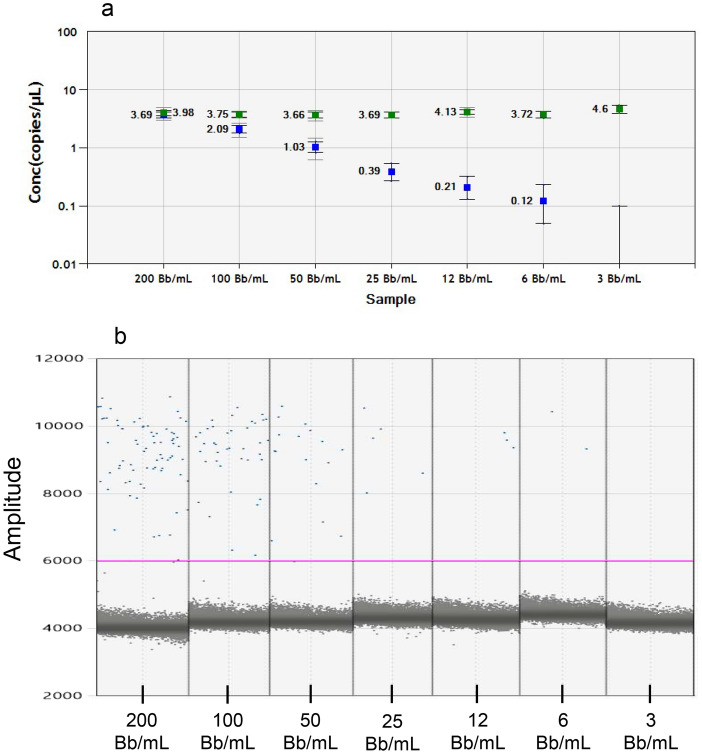
Evaluation of analytical sensitivity of the “direct” pre-PCR method on spiked samples in negative CSF matrix. *Bb Borrelia* bacteria. (**a**) Results given in copies/µL calculated by the QuantaSoft software. The blue squares represent the quantification of *Borrelia* copies, while the green squares represent the quantification of the internal positive control. (**b**) *Borrelia* results of the dilution series with different concentrations of spiked Bb in negative CSF matrix used for assessment of the analytical sensitivity. One representative output of each concentration is presented.

The analytical sensitivity of the optimized “direct” pre-PCR method was assessed using a dilution series of spiked *Borrelia* samples in the negative CSF matrix. The dilution series ranged from approximately 200 *Borrelia* targets per mL sample to 3 targets per mL sample. The analytical sensitivity was estimated to be approximately 10 *Borrelia* targets per mL sample, when using the lowest concentration (0.12 copies/µL/reaction) calculated by the QuantaSoft software (Fig. 4).

### Comparison of “direct” pre-PCR method to Maxwell DNA extraction method (C)

We performed a direct comparison of the optimised direct pre-PCR method to a commercial DNA extraction platform, the Maxwell purification system (Promega). Bb were spiked into either “negative CSF matrix” or the “CSF matrix with pleocytosis” and run in parallel using the optimised direct method or the Maxwell DNA extraction method as detailed in Supplementary C.

**Figure 5:**
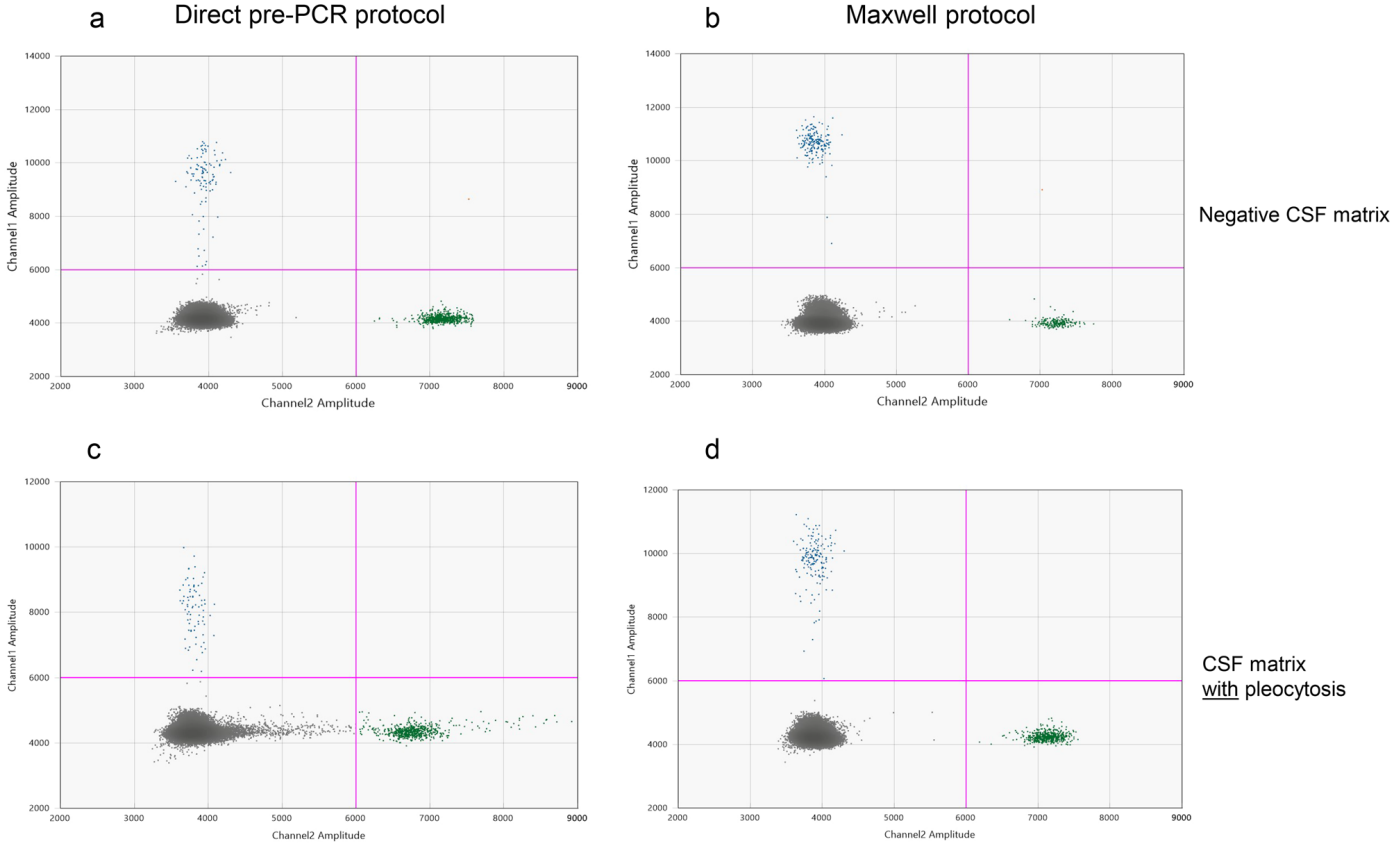
Comparison of “direct” pre-PCR method to commercial Maxwell DNA extraction method. The blue and green dots represent the *Borrelia* positive partitions and IPC positive partitions respectively, and grey dots represent the negative partitions. The horizontal pink lines illustrate the manual threshold setting for the data analysis. Amplitude on X and Y-axis refers to fluorescence intensity of individual partitions. For every spike experiment *Borrelia garinii* strain Lu118 was counted and diluted with either “negative CSF matrix” or “CSF matrix with pleocytosis”. Each figure shows the results of samples with a concentration of 50 Bb per mL. (**a**) “direct” pre-PCR method of samples in “negative CSF matrix”. (**b**) Maxwell purification of samples in “negative CSF matrix”, (**c**) “direct” pre-PCR method of samples in “CSF matrix with pleocytosis”. (**d**) Maxwell purification of samples in “CSF matrix with pleocytosis”.

**Figure 6:**
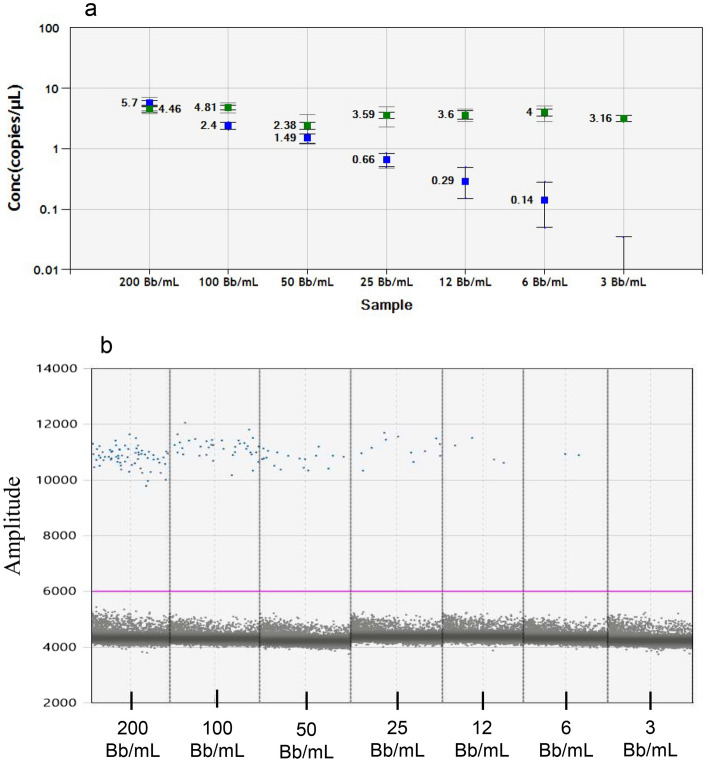
Evaluation of the analytical sensitivity of the Maxwell DNA extraction method. *Bb Borrelia* bacteria. (**a**) Results given in copies/µL calculated by the QuantaSoft software. The blue squares represent the quantification of *Borrelia* copies, while the green squares represent the quantification of the internal positive control. (**b**) *Borrelia* dPCR results of the dilution series with different concentrations of Bb spiked in negative CSF matrix used for assessment of the analytical sensitivity. One representative dPCR output of each concentration is presented.

The examined methods showed comparable results regarding total droplet counts and target quantification. However, the Maxwell extraction method demonstrated a better clustering of positive and negative partitions compared to the direct pre-PCR method, regarding both CSF matrix with and without pleocytosis (Fig. 5). Furthermore, the cellular components in the CSF matrix with pleocytosis did have a clear negative effect on PCR amplification of both the *Borrelia* and the IPC dPCR assays in the direct pre-PCR method compared to the Maxwell method, as indicated by the increase in amount of rain. The analytical sensitivity of the Maxwell method was evaluated to be approximately 10 *Borrelia* targets per mL sample, when using the lowest concentration (0.14 copies/µL) calculated by the QuantaSoft software (Fig. 6). Because of these results, we decided to continue the investigations of the clinical samples with the Maxwell purification method.

### *Borrelia* dPCR on patient samples

The clinical validation of the *Borrelia* dPCR method was performed on fifty-nine CSF samples from patients examined for LNB, nine patients with positive CSF/serum Bb-specific antibody index (AI) results and fifty patients with negative AI results and no CSF pleocytosis, respectively. All patient samples were centrifuged, and DNA was extracted using the Maxwell instrument, before dPCR analysis.

**Figure 7:**
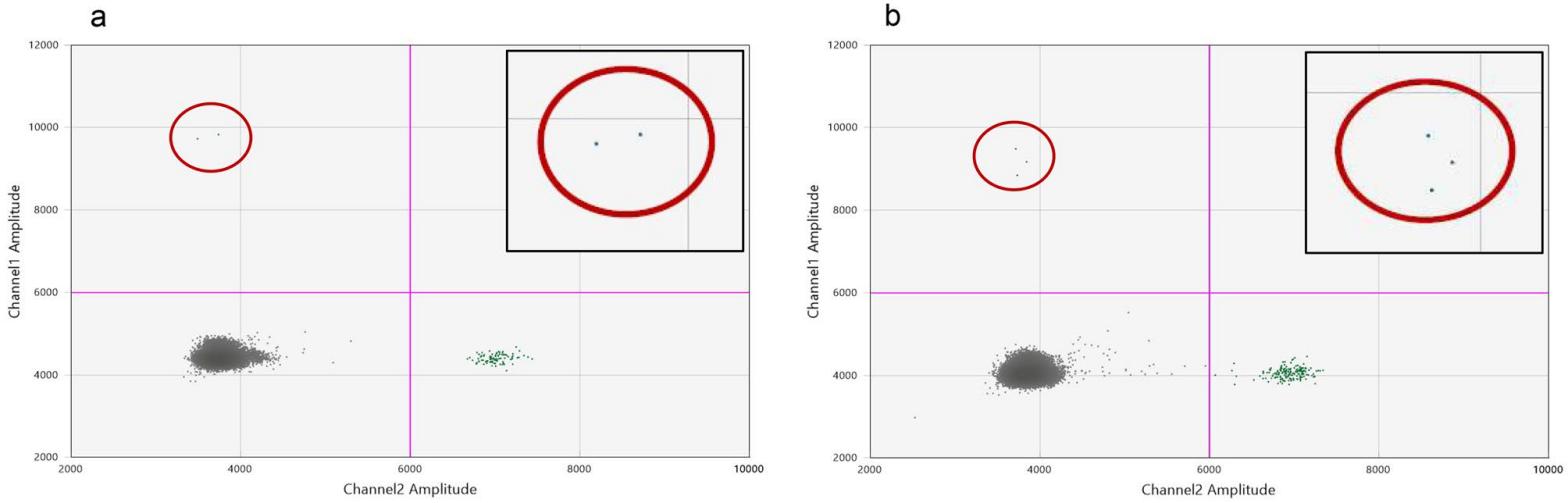
2D-dPCR output demonstrating the positive result a patient sample. (**a**) 1st dPCR run. (**b**) Repeat run. The blue and green dots represent the *Borrelia* positive partitions and IPC positive partitions respectively, and grey dots represent the negative partitions. The horizontal pink lines illustrate the manual threshold setting for the data analysis. Amplitude on X and Y-axis refers to fluorescence intensity of individual partitions. The red circles highlight the *Borrelia* positive partitions. The inserts visualize enlarged positive partitions.

The duration of illness, ages, and the laboratory findings of the nine LNB patients are presented in Table 1. One patient sample was positive for *Borrelia* DNA in the dPCR analysis. Thus, the clinical sensitivity was calculated to be 11.1% (1/9) and the clinical specificity was 100% (50/50). The patient with the positive *Borrelia* dPCR test had a short duration of symptoms (< 7 days) prior to lumbar puncture. The initial *Borrelia* dPCR result was confirmed by a repeat run on the same sample eluate. Both dPCR runs demonstrated very low amounts of *Borrelia* DNA in the patient sample (Fig. 7) corresponding to approximately 10 Bb per mL patient CSF sample.

**Table 1:** Clinical and laboratory findings in cerebrospinal fluid (CSF) samples from nine patients with Lyme neuroborreliosis.

Patient no.	Age (years)	Duration of illness^#^ (days)	CSF leukocyte counts (cells/µL)	Antibody index	CSF CXCL13 (pg/mL)	CSF volume for dPCR (mL)	CSF dPCR result
**1**	**10**	** < 7**	**140**	**IgM+**	**1394.6**	**0.6**	**Positive**
2	78	> 30	44	IgM+/IgG+	496.6	0.5	Negative
3	12	< 30	95	IgM+/IgG+	1304.5	0.7	Negative
4	69	> 30	180	IgG+	41.04	1	Negative
5	44	> 30	1200	IgM+/IgG+	2482.7	1	Negative
6	77	< 30	17	IgM+/IgG+	423.0	1	Negative
7	39	> 30	450	IgM+/IgG+	1026.1	1	Negative
8	47	> 30	220	IgM+/IgG+	1083.2	1	Negative
9	4	< 7	180	IgM+/IgG+	696.6	0.5	Negative

*dPCR* digital PCR.

^#^From symptom onset to lumbar puncture.

The patient with the positive dPCR result is highlighted in bold.

## Discussion

This study describes the experimental steps we have evaluated and implemented to ensure that the *Borrelia* dPCR method delivers the best possible performance, with emphasis on the given low Bb load in CSF samples. The results presented here, demonstrates that high analytical sensitivities are achievable using optimized methods (Figs. 4, 6). As presented, the different parameters that had the biggest impact on the performance of our *Borrelia* dPCR method were incubation of the PCR-plate at 4 °C prior to data acquisition, centrifugation, quick-spin, and matrix complexity. Our clinical validation of the optimized method delivers exploratory data from LNB patient samples with dPCR results, not impressive with one positive finding of nine intrathecal antibody confirmed LNB cases, but in concordance with previous studies.

In order to facilitate replication of our experiments, we have described the optimization process of our *Borrelia* dPCR assay method in details in compliance with updated *The Digital MIQE Guidelines*^22^. Subtleties to the dPCR application that are not necessarily obvious to new users of the technology, includes the impact of a temperature incubation step prior to droplet reading. The increase in droplet count after the overnight 4 °C hold is due to stabilization of the water-in-oil droplets. According to the manufacturer (personal communication with Bio-Rad) some droplets may shrink during PCR due to the evaporation of water and this is overturned by the cooling of the droplets. The size of the droplets is detected during the reading of the droplets and fluorescence signals from tiny droplets (assessed by a detector) will be dismissed by the Quantasoft software resulting in a reduced number of accepted droplets. A high number of accepted droplets is essential when detecting rare events, e.g., very few *Borrelia* bacteria, and therefore necessary for our experimental setup. However, a cooling step before droplet reading is not required in all dPCR applications.

By including an incubation step at 4 °C over-night of the PCR-plate prior to data acquisition, the overall method time is extended. This may be a disadvantage, when aiming to use the method for diagnosing LNB in the clinical laboratory. However, we consider that the benefit of maximizing the total droplet counts will greatly outweigh the disadvantage of increased turnaround time because more droplets read increases the chance of not missing the extremely rare *Borrelia* DNA targets in CSF.

In this study we examined the possibility of detecting *Borrelia* DNA by dPCR without the often-applied DNA extraction procedure prior to PCR analysis. This was done to avoid the theoretically loss of DNA material during purification procedures, and to exploit the ability of the dPCR analysis to amplify target molecules in reactions with PCR interfering components. To release the *Borrelia* DNA for detection, the spiked bacteria samples were subjected to simple boiling lysis. Our results confirm that *Borrelia* bacteria can be broken by boiling only, and that concentration of *Borrelia* targets by centrifugation is a central condition that improves the analytical sensitivity (Fig. 3). In line with our results, a recent study by Lager et al. showed that centrifugation of spiked samples significantly can improve the analytical sensitivity in terms of *Borrelia* bacteria per sample^20^. Centrifugation has also been suggested to influence the positive detection rate on clinical samples^8^. In our study, we also showed that a quick-spin step prior to dPCR reaction setup did improve cluster separation by pelleting the densest cellular debris (Fig. 3). Furthermore, we carried the quick-spin step on to the Maxwell method to ensure that residual magnetic beads would not interfere with droplet generation.

An important criterium of definite LNB diagnosis is CSF pleocytosis^11^. The cellular content of CSF samples may interfere with PCR amplification and is therefore an important parameter to evaluate when optimising the direct pre-PCR method. This became evident during the comparison of the direct pre-PCR method to the Maxwell purification method. Although we did not find any major effects of CSF pleocytosis on total droplet counts or target quantification, we did observe a poorer resolution between positive and negative partitions when comparing the two pre-analytical methods (Fig. 5). dPCR analysis on samples containing high amounts of target sequences will always result in some rain, and still provide acceptable results. However, when only one partitions have fluorescence amplitude above the negative cluster, results can be misclassified as negatives or interpreted as false positives. CSF samples with few *Borrelia* bacteria can therefore potentially result in ambiguous results, and subsequently repeat runs, due to intermediate fluorescence levels.

Our dPCR method was optimised using samples composed of negative CSF matrix spiked with known amounts of cultured *Borrelia* bacteria. We find this experimental setup to be the most suitable to evaluate how different pre-analytical conditions impact the analytical sensitivity and specificity. However, physiological differences between cultured *Borrelia* bacteria (laboratory adapted reference strains) and bacteria in clinical samples are evident, thus we also analysed patient CSF samples. We included fifty-nine patients examined for LNB, with samples stored at − 20 °C containing at least 0.5 mL CSF sample material. We detected *Borrelia* DNA in one clinical sample from a patient diagnosed with LNB resulting in a clinical sensitivity of 11% (1/9), which is within range of previous reports^9,10,23–25^. Positive PCR results for detection of *Borrelia* targets in CSF samples often have high cycle threshold values (> 38), indicating low amounts of targets in the samples^10,26^. The dPCR analysis of our positive patient sample resulted in similar weak signal with < 4 positive partitions per duplicate in each run (Fig. 7). We are aware that ideally this positive result should be verified by sequencing and that it is a limitation that we could not achieve that. However, given that the initial dPCR result is confirmed in a repeat run and that the clinical and laboratory findings support the data, we are confident that the dPCR positive results represents a true LNB case.

In general, the analytical sensitivity and specificity of PCR assays detecting *Borrelia* in CSF samples are high^9,26,27^, but the clinical sensitivity on CSF samples is suboptimal (median sensitivity of 40%)^25^. Consequently, it has been suggested that larger CSF sample volumes (> 1 mL) in combination with centrifugation may improve the possibility of detecting the *Borrelia* target by PCR^8,10,20,23^. A different approach enabling enrichment of *Borrelia* DNA prior to PCR could be to culture the clinical CSF samples. The turnaround time for *Borrelia* PCR results will increase significantly with this approach, however, the isolation and direct detection of the etiological agent from CSF samples will substantiate a clinical diagnosis of early LNB given patients with short duration of illness and initial negative *Borrelia*-specific AI results. Notably, we found one patient positive of the two patients with a duration of symptoms of less than a week indicating the importance of an early clinical suspicion of neuroborreliosis in patients with relevant symptoms.

In conclusion, our study has evaluated parameters important for *Borrelia*-specific dPCR assay performance and direct detection of *Borrelia* infection in CNS. The present study does not support the use of *Borrelia*-specific dPCR as a routine diagnostic method for diagnosing LNB. Future studies with focus on CSF pre-culture enrichment of *Borrelia* targets prior to PCR need to be performed during the earliest stage of a LNB possible.

## Materials and methods

### Experimental setups

An overview of the experimental workflow in this study is presented in Fig. 1 and the Supplementary Spreadsheet. A detailed description of each experiment is given in Supplementary A–C.

### DNA templates

#### Synthetic DNA fragments

Double-stranded DNA gBlock (Integrated DNA Technologies Inc., Iowa, USA) containing the target amplification sequence of the Bb-specific 16S rRNA gene (hereafter *Borrelia* gBlock) was initially used as positive PCR control during optimization of the in-house dPCR assay. Additionally, a gBlock containing a gene fragment of the Phocid herpesvirus (PhHV)^28^ was used as internal positive PCR control (IPC). Further details are provided in Supplementary A.

#### *Borrelia* strains

*Borrelia burgdorferi* sensu lato reference strains (Supplementary A, Table SA5) were used for optimization of pre-PCR procedures. Briefly described, *Borrelia* strains were cultured in 8 mL modified Kelly–Pettenkofer (MKP) medium at 35 °C under microaerophilic conditions^29^. The viability of the *Borrelia* spirochetes was assessed visually by dark-field microscopy (Leica DM2500, Triolab, Denmark) with 20× and 40× objectives. The Bb were counted using a Petroff–Hausser counting chamber (Cat. # 02-671-13, Thermo Fisher Scientific).

### Oligonucleotide information

The primers and probe for the *Borrelia* PCR assay was originally designed by Ornstein and Barbour to target a *Borrelia burgdorferi* sensu lato homogen-specific region of the 16S rRNA gene^30^. For the PhHV PCR assay, in-house validated forward and reverse primers were used together with a probe from the Wisselink study^28^. Table SA1 in Supplementary A details the primers and probes used in this study.

### Droplet digital PCR method

dPCR was performed on the QX100 Droplet Digital PCR System (Bio-Rad) throughout this study as per the manufacturer’s instructions, as described below and in the “Results” section. Details of the reagents, utensils and machinery are summarized in Table SA2, Supplementary A. dPCR run included a negative template control (NTC), and a positive PCR control, run in duplicates. PCR-grade water was used as NTCs. Negative CSF matrix (defined below) and Maxwell elution buffer were also used as negative controls in relevant experiments.

The preparation of the digital droplet PCR assay is given in details in Supplementary A. Described in brief, we assembled the reactions with the *Borrelia* and IPC oligonucleotides, ddPCR Supermix for probes (No dUTP) (Bio-Rad 186-3023), and DNA template to a final volume of 22.0 μL (10% excess) and transferred them to a 96-well plate. Droplets were produced on the Automated Droplet Generator (Bio-Rad) from 20 μL of the prepared reaction. The PCR plate was sealed (Bio-Rad) and PCR amplification was performed with the Veriti thermal cycler (Applied Biosystems). The PCR cycling conditions are given in Table SA3, Supplementary A. Following final hold, the partitions were read on the QX100 Droplet reader (Bio-Rad), and data was acquired using the QuantaSoft v.1.7.4.0917 software. The partitions were classified as positives or negatives using manual threshold settings guided by fluorescence amplitudes of both the positive controls and the NTC. For each reaction, the droplet count should be of at least 15,000 droplets to be accepted otherwise the sample was re-run.

#### Verification of Borrelia dPCR assay specificity and sensitivity

Quality Control for Molecular Diagnostics (QCMD) past panels (Qnostics) and cultured *Borrelia burgdorferi* sensu lato strains were used for inclusivity/exclusivity experiments. The content of QCMD CNS 2017 + 2018 and ME 2017 past panels and the Bb reference strains are presented in Supplementary A, Tables SA4 and SA5.

### Cerebrospinal fluid matrices for spike experiments

Anonymised CSF specimens from 150 patients *withou*t CSF pleocytosis (< 5 cells/µL) and non-infectious CNS diseases, were pooled and used as CSF spike matrix for optimization experiments, hereafter designated “negative CSF matrix”. To ensure that the CSF spike matrix was negative for CNS disease causing microorganisms, the negative CSF matrix was examined by the FilmArray Meningitis/Encephalitis (ME) PCR Panel (BioFire Diagnostics, Salt Lake City, Utah, USA).

For evaluation of the effect of CSF pleocytosis on dPCR interference, anonymised CSF specimens from patients *with* CSF pleocytosis (> 4 cells/µL) were pooled and diluted to contain approximately 100 cells/µL and designated “CSF pleocytosis matrix” hereafter.

### Maxwell DNA extraction

The Maxwell® 16 Cell LEV DNA Purification Kit (Promega, Madison, Wisconsin, USA) was used with the automated Maxwell® 16 Instrument (Promega) configured with the low elution volume (LEV) hardware according to the manufacturer’s instructions. Samples (100 µL) was subjected to the purification kit together with 100 µL elution buffer.

### Patient samples

CSF sample aliquots from patients examined for LNB at the Department of Clinical Microbiology Lillebaelt Hospital in the Region of Southern Denmark were included in this study. All samples were sent to the laboratory from October 2019 to March 2021 as part of routine LNB diagnosis and CSF aliquots were stored at − 20 °C until dPCR analysis. Patient aliquots were only analysed by the *Borrelia* dPCR method if they contained ≥ 0.5 mL CSF. The need for patient approval and informed consent was waived by the ethics committee of the institutional review board at Lillebaelt Hospital.

There were nine CSF samples from patients diagnosed with definite LNB, defined by a positive CSF/serum Bb-specific antibody index (AI) (IDEIA Lyme Neuroborreliosis test, Oxoid, Hampshire, UK), neurological symptoms consistent with LNB, and concurrent CSF pleocytosis. Patient information provided by the electronic medical record and the local electronic laboratory data management systems for clinical biochemistry (BCC) and microbiology (MADS) included duration of illness, antibiotic treatment prior to lumbar puncture, CSF leukocyte counts, CSF/serum Bb-specific AI results and CSF CXCL13 quantities. As controls we used CSF samples that tested negative in the CSF/serum Bb-specific AI, designated the non-LNB patients.

### Data analysis

All data analysis was done using the QuantaSoft™ v.1.7.4.0917 and the QuantaSoft™ Analysis Pro 1.0.596 software. Figures and tables were made in Word and Excel v. 2102 (Microsoft Corporation, USA).

### Ethics statement

Collection and use of data for this study were conducted in accordance with the declaration of Helsinki and was approved as a quality development project by the ethics committee of the institutional review board at Lillebaelt Hospital (18/62866). This study was registered as public research of the Region of Southern Denmark according to the General Data Protection Regulation (18/63046).

## Supplementary Information

Supplementary Information.

## Data Availability

Data is available upon reasonable request after contact with the corresponding author.

## References

[1] Stanek G (2011). Lyme borreliosis: Clinical case definitions for diagnosis and management in Europe. Clin. Microbiol. Infect..

[2] Strle F, Stanek G (2009). Clinical manifestations and diagnosis of Lyme borreliosis. Curr. Probl. Dermatol..

[3] Hansen K, Lebech AM (1992). The clinical and epidemiological profile of Lyme neuroborreliosis in Denmark 1985–1990. A prospective study of 187 patients with *Borrelia burgdorferi* specific intrathecal antibody production. Brain.

[4] Stanek G, Strle F (2018). Lyme borreliosis-from tick bite to diagnosis and treatment. FEMS Microbiol. Rev..

[5] Stanek G, Reiter M (2011). The expanding Lyme Borrelia complex—Clinical significance of genomic species?. Clin. Microbiol. Infect..

[6] Strle F, Ruzic-Sabljic E, Cimperman J, Lotric-Furlan S, Maraspin V (2006). Comparison of findings for patients with *Borrelia garinii* and *Borrelia afzelii* isolated from cerebrospinal fluid. Clin. Infect. Dis..

[7] Lebech AM, Hansen K, Brandrup F, Clemmensen O, Halkier-Sørensen L (2000). Diagnostic value of PCR for detection of *Borrelia burgdorferi* DNA in clinical specimens from patients with erythema migrans and Lyme neuroborreliosis. Mol. Diagn..

[8] Barstad B (2018). Direct molecular detection and genotyping of *Borrelia burgdorferi* sensu lato in cerebrospinal fluid of children with Lyme neuroborreliosis. J. Clin. Microbiol..

[9] de Leeuw BH (2014). Evaluation of Borrelia real time PCR DNA targeting OspA, FlaB and 5S–23S IGS and Borrelia 16S rRNA RT-qPCR. J. Microbiol. Methods.

[10] Forselv KJN (2018). Does more favourable handling of the cerebrospinal fluid increase the diagnostic sensitivity of *Borrelia burgdorferi* sensu lato-specific PCR in Lyme neuroborreliosis?. Infect. Dis. (Lond.).

[11] Mygland A (2010). EFNS guidelines on the diagnosis and management of European Lyme neuroborreliosis. Eur. J. Neurol..

[12] Ljøstad U, Skarpaas T, Mygland A (2007). Clinical usefulness of intrathecal antibody testing in acute Lyme neuroborreliosis. Eur. J. Neurol..

[13] Ružić-Sabljić E, Cerar T (2017). Progress in the molecular diagnosis of Lyme disease. Expert Rev. Mol. Diagn..

[14] Huggett JF, Whale A (2013). Digital PCR as a novel technology and its potential implications for molecular diagnostics. Clin. Chem..

[15] Whale AS (2012). Comparison of microfluidic digital PCR and conventional quantitative PCR for measuring copy number variation. Nucleic Acids Res..

[16] Dingle TC, Sedlak RH, Cook L, Jerome KR (2013). Tolerance of droplet-digital PCR vs real-time quantitative PCR to inhibitory substances. Clin. Chem..

[17] Vynck M, Vandesompele J, Thas O (2018). On determining the power of digital PCR experiments. Anal. Bioanal. Chem..

[18] King JL, Smith AD, Mitchell EA, Allen MS (2017). Validation of droplet digital PCR for the detection and absolute quantification of Borrelia DNA in Ixodes scapularis ticks. Parasitology.

[19] Das S, Hammond-McKibben D, Guralski D, Lobo S, Fiedler PN (2020). Development of a sensitive molecular diagnostic assay for detecting *Borrelia burgdorferi* DNA from the blood of Lyme disease patients by digital PCR. PLoS ONE.

[20] Lager M, Wilhelmsson P, Matussek A, Lindgren P-E, Henningsson AJ (2021). Molecular detection of Borrelia bacteria in cerebrospinal fluid-optimisation of pre-analytical sample handling for increased analytical sensitivity. Diagnostics.

[21] Karlin S, Altschul SF (1990). Methods for assessing the statistical significance of molecular sequence features by using general scoring schemes. Proc. Natl. Acad. Sci..

[22] Huggett JF (2013). The digital MIQE guidelines: Minimum information for publication of quantitative digital PCR experiments. Clin. Chem..

[23] Skogman BH (2021). Lyme neuroborreliosis in Swedish children—PCR as a complementary diagnostic method for detection of *Borrelia burgdorferi* sensu lato in cerebrospinal fluid. Eur. J. Clin. Microbiol. Infect. Dis..

[24] Cerar T (2008). Validation of cultivation and PCR methods for diagnosis of Lyme neuroborreliosis. J. Clin. Microbiol..

[25] Dessau RB (2018). To test or not to test? Laboratory support for the diagnosis of Lyme borreliosis: A position paper of ESGBOR, the ESCMID study group for Lyme borreliosis. Clin. Microbiol. Infect..

[26] Gooskens J, Templeton KE, Claas EC, Van Dam AP (2006). Evaluation of an internally controlled real-time PCR targeting the ospA gene for detection of *Borrelia burgdorferi* sensu lato DNA in cerebrospinal fluid. Clin. Microbiol. Infect..

[27] Lebech AM, Hansen K (1992). Detection of *Borrelia burgdorferi* DNA in urine samples and cerebrospinal fluid samples from patients with early and late Lyme neuroborreliosis by polymerase chain reaction. J. Clin. Microbiol..

[28] Wisselink GJ, van Zanten E, Kooistra-Smid AM (2011). Trapped in keratin; a comparison of dermatophyte detection in nail, skin and hair samples directly from clinical samples using culture and real-time PCR. J. Microbiol. Methods.

[29] Wagemakers A, Oei A, Fikrig MM, Miellet WR, Hovius JW (2014). The relapsing fever spirochete *Borrelia miyamotoi* is cultivable in a modified Kelly-Pettenkofer medium, and is resistant to human complement. Parasit. Vectors.

[30] Ornstein K, Barbour AG (2006). A reverse transcriptase-polymerase chain reaction assay of *Borrelia burgdorferi* 16S rRNA for highly sensitive quantification of pathogen load in a vector. Vector Borne Zoonotic Dis..

