# Supplementary material for: Establishment of a digital PCR method for detection of Borrelia burgdorferi sensu lato complex DNA in cerebrospinal fluid
Source: Sci Rep. 2022 Nov 21;12:19991. doi: 10.1038/s41598-022-24041-8 (PMC9678864; doi:10.1038/s41598-022-24041-8)
Supplement: Supplementary file 1 — Supplementary Information. [file 41598_2022_24041_MOESM1_ESM.pdf]

SUPPLEMENTARY

Establishment of a digital PCR method for detection of *Borrelia burgdorferi* sensu lato complex DNA in cerebrospinal fluid

AUTHORS

Trine Andreasen Leth*[1,2], Sara Moeslund Joensen[1], Malene Bek-Thomsen[3] and Jens Kjølseth Møller[1,2]

[1] *Department of Clinical Microbiology, Lillebaelt Hospital – University Hospital of Southern Denmark*

[2] *Department of Regional Health Research, Faculty of Health Sciences, University of Southern Denmark*

[3] *Department of Clinical Genetics, Aarhus University Hospital, Denmark*

SUPPLEMENTARY - A

This section details the establishment of the dPCR assay in the first stage; Establishment of *Borrelia* dPCR assay (A). A simplified overview of all experimental setup is presented in the supplemental spreadsheet.

METHODS in details

Synthetic DNA gBlocks

gBlocks were provided in dry form and subsequently resuspended in high-quality molecular graded water containing 0.1 mg/mL Brewer's yeast tRNA (Roche) to a stock concentration of 10 ng/µL. Initially, the stock concentrations were converted to copy number concentration using the stock concentrations, the molecular weight of the gBlocks and Avogradro's number (6.022×10^{23}). However, later the QX100™ Droplet Digital PCR System (Bio-Rad) was used to determine the copy number concentration using the in-house assays. An eleven-point ten-fold serial volumetric dilutions of the stock DNA gBlock into molecular graded water containing 0.1 mg/mL Brewer's yeast tRNA were performed to produce five 50 µL triple use aliquots containing ~120 copies/µL. All aliquots were stored at -20 °C and were stable for a period of at least 3 months. An aliquot was thawed maximum 3 times and used without further dilution. The *Borrelia* and the IPC gBlock sequences are provided in supplemental Table SA1 below.

Primers and probes

All primers and probes were manufactured by LGC Biosearch Technologies (Risskov, Denmark) and were shipped in dry form. The oligonucleotides were resuspended in high-quality molecular graded water to stock concentrations of 100 µM for long term storage in -20 °C freezer and to working stocks with concentrations of 20 µM for short term use with 3-5 freeze-thaw cycles.

All primer and probe sequences are provided in supplemental Table SA1.

Assay	Length	Tm °C	Sequence	Reference
Borrelia				**Ornstein and Barbour 2006.**
Forward	22	58.2	5'-GGTCAAGACTGACGCTGAGTCA-3'	
Reverse	22	58	5'-GGCGGCACACTTAACACGTTAG-3'	
Probe	29	61.7	5'-6FAM-TCTACGCTGTAAACGATGCACACTTGGTG-BHQ1-3'	
Borrelia gBlock	204		5'-CAGAAAGAATACCGGAGGCGAAGGCGAACTTCTGGGTCAAGACTGACGCTGAGTCACGAAAGCGTAGGGAGCAAACAGGATTAGATACCCTGGTAGTCTACGCTGTAAACGATGCACACTTGGTGTTAACTAAAGTTAGTACCGAAGCTAACGTGTTAAGTGTGCCGCCTGGGGAGTATGCTCGCAAGAGTGAAACTCAAAGG-3'	
**IPC**				
Forward	26	53.4	5'-AAGCGTGATATTGCTCTTTCGTATAG-3'	**This article**
Reverse	30	53.3	5'-ACATAGCGACAGATTACAACATTAGTATTG-3'	**This article**
Probe	28	60	5'-HEX-TTTTTATGTGTCCGCCACCATCTGGATC-BHQ1-3'	**Wisselink et al. 2011**
PhHV gBlock	172		5'-GATGATATGAGAGAAATTTTGGGCGAATCACAGATTGAATCTTAAGCGTGATATTGCTCTTTCGTATAGATTTTTTATGTGTCCGCCACCATCTGGATCAACAATACTAATGTTGTAATCTGTCGCTATGTGGTTGGTACGTTTGGAACCGCCTCGGGCTTGTCCTAATTAC-3'	

Table SA1. Primers, probes and gBlocks included in the dPCR analysis. The *Borrelia* primers and probe targets a *Borrelia burgdorferi* sensu lato specific region of the 16S rRNA gene and was originally designed by Ornstein and Barbour in 2006. The IPC PCR assay is an in-house validated assay used for control of PCR amplification. The underlined sequence of the *Borrelia* gBlock represents the 136 base pair amplicon of the *Borrelia* dPCR assay. The underlined sequence of the PhHV gBlock represents the 88 base pair amplicon of the IPC dPCR assay.

Digital droplet PCR assay assembly

The full list of reagents, utensils and instruments used in the dPCR experiments is given in supplemental Table SA2 below. The dPCR reactions were prepared in a dedicated pre-PCR room. First, the dPCR Supermix enzyme was mixed thoroughly with water and the primers and probes by

pulse-vortexing the mixture five times for 5-10 sec followed by a quick-spin of 10 sec. The PCR-mixture was then distributed across the relevant number of Eppendorf tubes (Sarstedt), one for each sample to be tested in duplicates. 11.5 µL *Borrelia* DNA target (gBlock or bacteria) or the positive PCR control were added to the relevant tubes together with 1 µL IPC gBlock (concentration of ~120 copies/µL), which corresponds to 5 µL target per 20 µL PCR mix. The NTCs were made with PCR-grade water. All the PCR-samples with a total volume of 50 µL were then mixed thoroughly again by pulse-vortexing the mixture five times for 5-10 sec followed by a quick-spin of 10 sec. For each PCR-reaction, 22 µL of each PCR-sample was transferred to a well on a semi-skirted 96 well PCR-plate (Eppendorf). 1x dPCR buffer control was used in empty wells on the PCR-plate so that any unused wells were filled prior to droplet generation. The PCR-plate was sealed and placed in the Auto DG droplet generator (Bio-Rad). An empty PCR-plate was placed on a cooling element in the Auto DG. After droplet generation the newly filled PCR-plate was immediately sealed and subsequently placed in the Veriti Thermal cycler for PCR amplification. The PCR-program is summarized in supplemental Table SA3. Following PCR and the over night final hold at 4 °C the PCR-reaction are read by the QX100 Reader with chosen FAM/HEX channels.

**Component**	**Vendor**	**Item #**
ddPCR™ Supermix for Probes (No dUTP)	Bio-Rad	1863023
Primers	LGC Biosearch Technologies	Custom
Probes	LGC Biosearch Technologies	Custom
PCR-grade Water	Sigma-Aldrich	W4502-1L
Borrelia gBlock	Integrated DNA Technologies	Custom
PhHV IPC gBlock	Integrated DNA Technologies	Custom
Automated Droplet Generation Oil for Probes	Bio-Rad	1864110
ddPCR™ droplet Reader oil	Bio-Rad	1863004

ddPCR™ Buffer control kit for probes	Bio-Rad	1863052
DG32 Automated Droplet Generator Cartridge	Bio-Rad	1864108
Pierceable Foil Heat seal	Bio-Rad	1814040
Eppendorf twin.tec® PCR-plate 96, green	VWR, Avantor	3912051
Eppendorf twin.tec® PCR-plate 96, red	VWR, Avantor	3912053
Sartorius, 1000 µL pipette tips	Dandiag	791001F
Sartorius, 300 µL pipette tips	Dandiag	790301F
Sartorius, 120 µL pipette tips	Dandiag	790101F
Sartorius, 10 µL pipette tips	Dandiag	790011F
QX200 Auto DG	Bio-Rad	
PX1	Bio-Rad	
Veriti Thermal cycler	Applied Biosystems, Thermo Fisher Scientific	
QX100 Reader	Bio-Rad	

Table SA2. List that summarizes the reagents, utensils, and instruments used for the dPCR experiments.

**Step**	**Temperature**	**Time**		**Cycles**
Polymerase activation	95 °C	10 min		1
Denaturation	95 °C	30 sec	}	40
Annealing + extension	62 °C	60 sec		
Droplet stabilization	98 °C	10 min		1
Final hold	4 °C	Over night		

Table SA3. PCR thermal cycling conditions of the dPCR analysis. All ramp rates were set to 2 °C/second. The over night final hold at 4 °C was implemented in the PCR-program because of the empirically evaluated effect on the total droplet counts.

Borrelia specificity

Borrelia dPCR assay specificity was evaluated using purified DNA from the following pathogens provided by QCMD past panels (Table SA4) and purified DNA from *Borrelia* reference strains (Table SA5).

**Microorganism**	
**Bacteria**	
Escherichia coli	QCMD ME17 past panel + culture strain
Haemophilus influenzae	QCMD ME17 past panel
Listeria monocytogenes	QCMD ME17 past panel + culture strain
Neisseria meningitidis	QCMD ME17 past panel
Streptococcus agalactiae	QCMD ME17 past panel
Streptococcus pneumoniae	QCMD ME17 past panel + culture strain
**Virus**	
Enterovirus	QCMD CNS17 past panel
Herpes simplex-virus 1	QCMD CNS17 and CNS18 past panels
Herpes simplex-virus 2	QCMD CNS17 and CNS18 past panels
Human parechovirus	QCMD CNS17 and CNS18 past panels
Varicella zoster-virus	QCMD CNS17 and CNS18 past panels
Echovirus	QCMD CNS18 past panel
JC virus	QCMD CNS17 and CNS18 past panels
**Yeast**	
Cryptococcus neoformans	QCMD ME17 past panel

Table SA4. The content of QCMD CNS 2017+2018 and ME 2017 past panels.

***Borrelia burgdorferi* sensu lato species**	**Genotype**	**Provider**
B. afzelii	BO23	ATCC-51992
B. burgdorferi sensu stricto	B31	ATCC-35210
B. bavariensis	Pbi	ATCC-BAA-2496
B. garinii	CIP 103362	ATCC-51383
B. garinii	Phei	Volker Fingerle
B. burgdorferi sensu stricto	B31 wt	Sven Bergström via Malin Lager
B. burgdorferi sensu stricto	B313	Sven Bergström via Malin Lager
B. garinii	Lu118	Sven Bergström via Malin Lager
B. garinii	Lu190	Sven Bergström via Malin Lager
B. afzelii	Lu171	Sven Bergström via Malin Lager
B. afzelii	Lu207	Sven Bergström via Malin Lager

Table SA5. List of *Borrelia burgdorferi* sensu lato reference strains.

Supplemental RESULTS

Forward primer (nM)	Reverse primer (nM)	Mean positive amplitude	SD	Mean negative amplitude	SD	Mean amplitude difference
300	300	7010	74	3292	53	3718
300	600	6652	93	2946	119	3707
300	900	6336	72	2657	49	3679
300	1200	6936	32	2984	60	3952
600	300	7041	40	2932	39	4109
600	600	7960	98	3164	64	4797
600	900	6860	29	2686	137	4174
600	1200	6807	104	2568	221	4239
900	300	7413	110	3067	42	4346
900	600	7625	136	3000	113	4626
**900**	**900**	**8111**	**37**	**3111**	**15**	**5001**
900	1200	7920	104	3008	32	4912
1200	300	8068	131	3443	40	4625
1200	600	7399	26	2878	13	4521
1200	900	7432	61	2763	28	4669
1200	1200	7032	155	2457	47	4575

Table SA6. Results of the evaluated primer matrices for assessment of optimal combination of forward and reverse primer concentrations. The best combination of forward and reverse primer concentrations is highlighted in bold. SD = standard deviation.

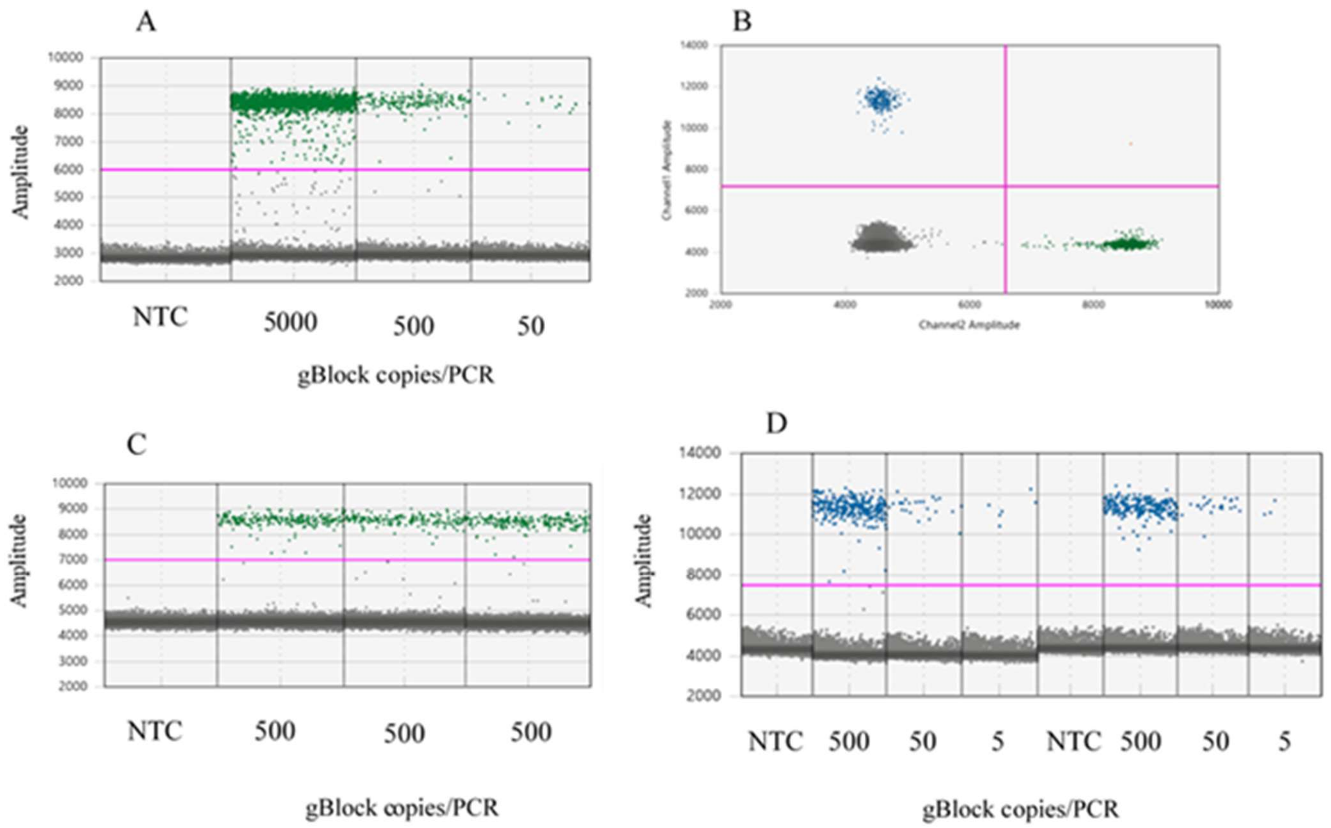

Figure SA1. Examples of dPCR output after the addition of the IPC (internal positive control) dPCR assay to the *Borrelia* dPCR assay. Each column represents samples from individual wells and is separated by a grey line. Blue dots represent the positive droplets and grey dots represent the negative droplets. The horizontal pink lines illustrate the manual threshold setting for the data analysis. Amplitude on Y-axis refers to fluorescence intensity of individual droplets. NTC (non-template control) was molecular graded water in all experiments. (A) Illustrates the IPC dPCR assay at different IPC gBlock concentrations. (B). The 2D-dPCR output of the *Borrelia* and IPC dPCR duplex. (C+D) Illustrates the *Borrelia*/IPC dPCR duplex and effect of 500 copies/PCR IPC gBlock on the *Borrelia* assay. In D the first 4 wells are the Borrelia dPCR singleplex assay, and the last 4 wells are the Borrelia/IPC duplex.

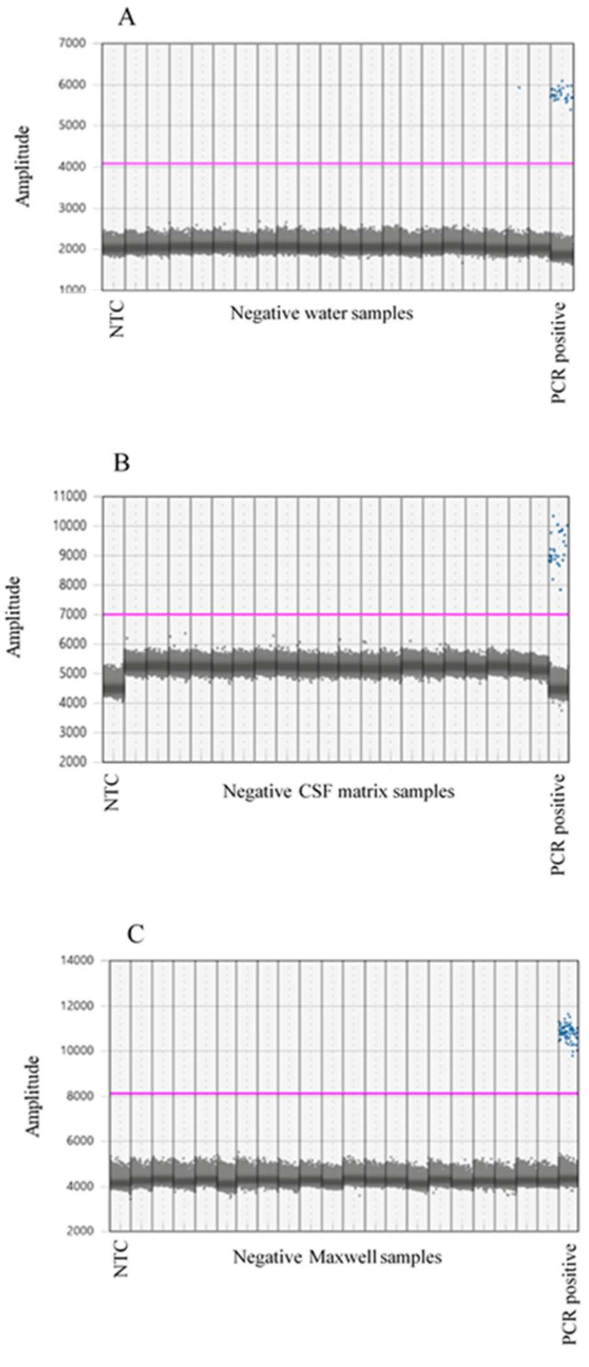

Figure SA2. Evaluation of dPCR false-positives rate of water, negative CSF matrix and Maxwell elution buffer in the dPCR duplex. Each column represents samples from individual wells and is separated by a grey line. Blue dots represent the positive droplets and grey dots represent the negative droplets. The horizontal pink lines illustrate the manual threshold setting for the data analysis. Amplitude on Y-axis refers to fluorescence intensity of individual droplets. NTC (non-template control) was molecular graded water in all experiments. (A) dPCR results from samples with only water. (B) dPCR results from samples with the negative CSF matrix. (C) dPCR results from samples with Maxwell elution buffer.

SUPPLEMENTARY - B

Detailed information regarding each experimental setup in the second stage; Optimization of a "direct" pre-PCR method without DNA extraction (B). A simplified overview of all experimental setup is presented in Supplementary spreadsheet.

The samples were evaluated as freshly spiked samples in each setup. No storing or freeze/thaw cycles were applied.

I. Boiling (*Borrelia* bacteria lysis)

- A dilution series ranging from 10,000 to 100 bacteria mL^{-1} of *B. garinii* strain Lu118 was prepared. By applying 100 µL per dilution to 900 µL negative CSF matrix, a final concentration of 10 to 1000 Bb per sample were achieved. Each concentration was examined in triplicates.
- A negative control consisting of negative CSF matrix was included in the experiment.
- Each sample was placed onto a pre-warmed block heater set to 95 °C for 10 min. followed by cooling at 4 °C for 10 min in the fridge.
- All samples were pulse-vortexed five times for 5 sec. prior to dPCR analysis. The dPCR assembly and analysis is described in Supplementary A.
- A flowchart of the procedure is given in Figure SB1 below.

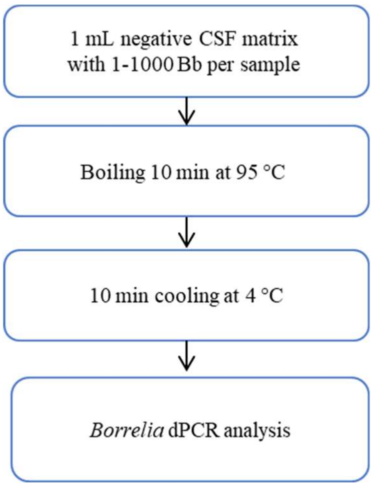

Figure SB1. Flowchart illustrating the Bb lysis experiment by boiling the samples prior to digital droplet PCR analysis. CSF = cerebrospinal fluid. Bb = *Borrelia* bacteria. dPCR = digital droplet PCR.

II. Centrifugation (*Borreli*a bacteria concentration)

- A dilution series ranging from 10,000 to 100 bacteria mL^{-1} of *B. garinii* strain Lu118 was prepared. By applying 100 µL per dilution to 900 µL negative CSF matrix, a final concentration of 10 to 1000 Bb per sample were achieved. Each concentration was examined in triplicates.
- A negative control consisting of negative CSF matrix was included in the experiment.
- All samples were placed in an Eppendorf MiniSpin benchtop centrifuge and rotated with 14,000 xg for 20 min. Afterwards 900 µL supernatant was discarded by manually pipetting technique, and the remaining 100 µL supernatant and pelleted material was mixed by pulse-vortexing the sample five times 5-10 sec.
- Each sample was then placed onto a pre-warmed block heater set to 95 °C for 10 min. followed by cooling at 4 °C for 10 min in the fridge.
- The subsequently dPCR assembly and analysis followed the description in Supplementary A.
- A flowchart of the procedure is given in Figure SB2 below.

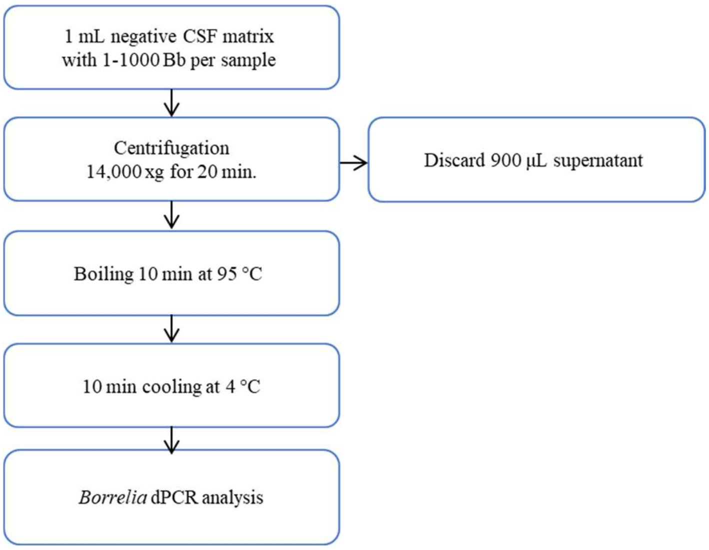

Figure SB2. Flowchart illustrating the Bb concentration experiment by centrifugation of the samples prior to digital droplet PCR analysis. CSF = cerebrospinal fluid. Bb = *Borrelia* bacteria. dPCR = digital droplet PCR.

III. Quick-spin (reduction of dPCR rain)

- A dilution series ranging from 10,000 to 100 bacteria mL^{-1} *B. garinii* strain Lu118 was prepared. By applying 100 µL per dilution to 900 µL negative CSF matrix, a final concentration of 10 to 1000 Bb per sample were achieved. Each concentration was examined in duplicates.
- A negative control consisting of negative CSF matrix was included in the experiment.
- All samples were placed in an Eppendorf MiniSpin benchtop centrifuge and rotated with 14,000 xg for 20 min. Afterwards 900 µL supernatant was discarded by manually pipetting technique, and the remaining 100 µL supernatant and pelleted material was mixed by pulse-vortexing the sample five times 5-10 sec.
- Each sample was then placed onto a pre-warmed block heater set to 95 °C for 10 min. followed by cooling at 4 °C for 10 min in the fridge.
- The samples were all subjected to quick-spin by Prism mini centrifuge for 5 sec. at 2000 xg. The subsequently dPCR assembly and analysis followed the description in Supplementary A.
- A flowchart of the procedure is given in Figure SB3 below.

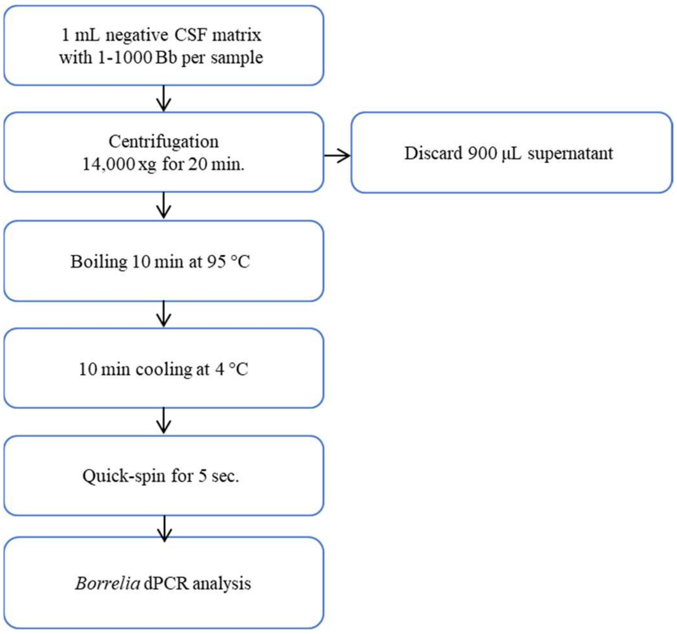

Figure SB3. Flowchart illustrating the placement of the quick-spin step prior to digital droplet PCR analysis. CSF = cerebrospinal fluid. Bb = *Borrelia* bacteria. dPCR = digital droplet PCR.

IV. Proteinase K treatment (*Borrelia* bacteria lysis and reduction of dPCR rain)

- A dilution series ranging from 10,000 to 100 bacteria mL^{-1} of *B. garinii* strain Lu118 was prepared. By applying 100 µL per dilution to 900 µL negative CSF matrix, a final concentration of 10 to 1000 Bb per sample were achieved. Each concentration was examined in duplicates.
- A negative control consisting of negative CSF matrix was included in the experiment.
- All samples were placed in an Eppendorf MiniSpin benchtop centrifuge and rotated with 14,000 xg for 20 min. Afterwards 900 µL supernatant was discarded by manually pipetting technique, and the remaining 100 µL supernatant and pelleted material was mixed by pulse-vortexing the sample five times 5-10 sec.
- Each sample was then placed onto a pre-warmed block heater set to 95 °C for 10 min. followed by cooling at 4 °C for 10 min in the fridge.
- The samples were all subjected to further lysis by adding 20 µL proteinase K (Promega) to the sample vials, brief vortexing, followed by incubation at 56 °C for 1 h and Proteinase K inactivation at 95 °C for 10 min. The subsequently dPCR assembly and analysis followed the description in Supplementary A.
- A flowchart of the procedure is given in Figure SB4 below.

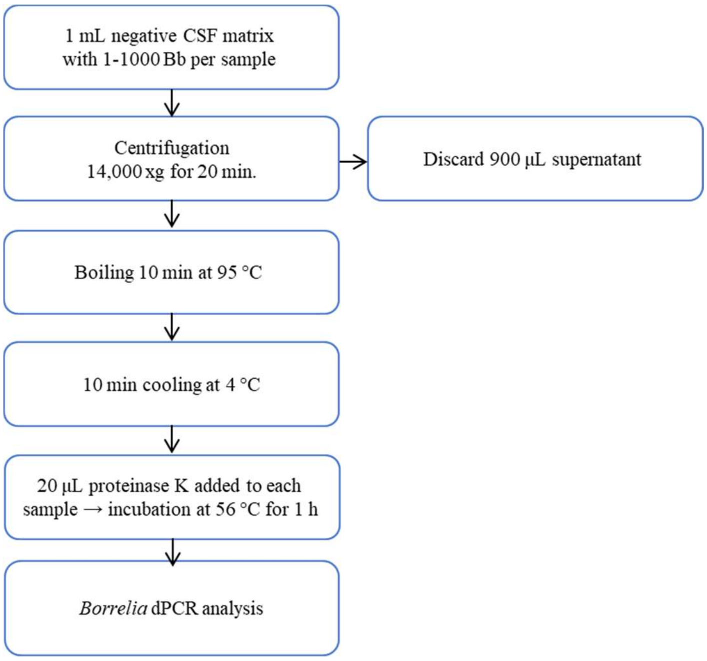

Figure SB4. Flowchart showing the proteinase K treatment step prior to digital droplet PCR analysis. CSF = cerebrospinal fluid. Bb = *Borrelia* bacteria. dPCR = digital droplet PCR.

SUPPLEMENTARY - C

Detailed information regarding the experimental setup in the third stage; Comparison of "direct" pre-PCR method to Maxwell DNA extraction method (C). A simplified overview of all experimental setup is presented in the supplemental spreadsheet. The samples were evaluated as freshly spiked samples.

Comparison of "direct" pre-PCR method to Maxwell DNA extraction method (C)

- Dilutions of 50 cells of *B. garinii* strain Lu118 mL^{-1} were prepared in either "negative CSF matrix" or "CSF matrix with pleocytosis".
- Negative controls consisting of empty matrix samples were included in each setup.
- All samples were placed in a benchtop centrifuge and rotated with 14,000 xg for 20 min. Afterwards 900 µL supernatant was discarded by manually pipetting technique, and the remaining 100 µL supernatant and pelleted material was mixed by pulse-vortexing the sample five times 5-10 sec.
- The samples were then either subjected to the direct pre-PCR method including boiling and colling procedures (described in Supplementary B) or to automated DNA extraction on the Maxwell platform.
- All samples were then subjected to quick-spin by Prism™ mini centrifuge for 5 sec. at 2000 xg. The subsequently dPCR assembly and analysis followed the description in Supplementary A.
- A flowchart of the experimental setup is given in Figure SC1 below.

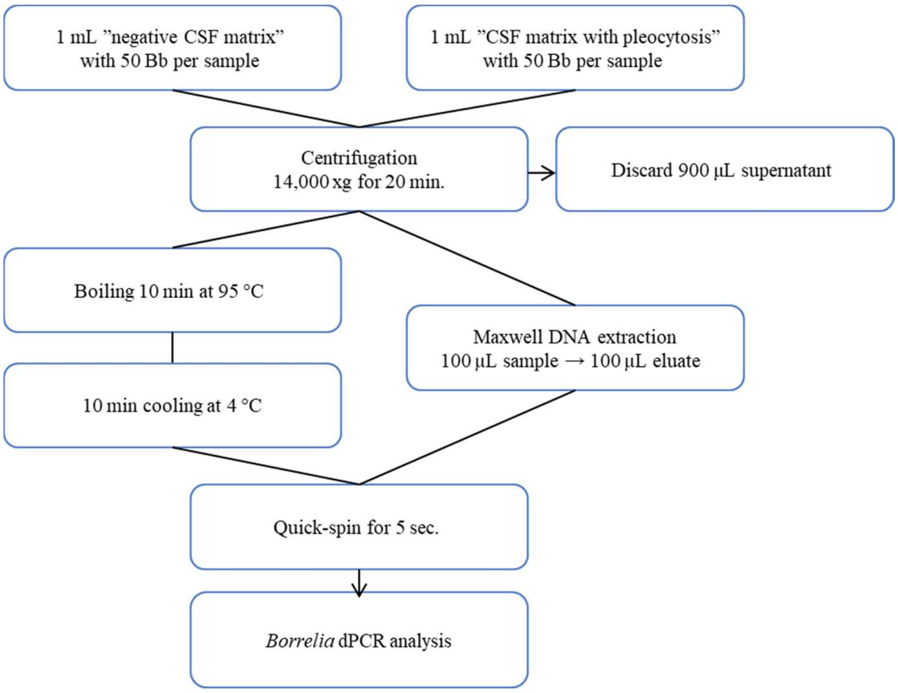

Figure SC1. Flowchart illustrating the placement of the quick-spin step prior to digital droplet PCR analysis. CSF = cerebrospinal fluid. Bb = *Borrelia* bacteria. dPCR = digital droplet PCR.

Supplementary speadsheet to “Establishment of an in-house digital PCR method for detection of *Borrelia burgdorferi* sensu lato complex bacteria DNA in cerebrospinal fluid specimens”

Overview of different stages that were performed

**Experiemental setup**		**Parameter/purpose**	**DNA target**	**Concentration range of target**	**Fluid**	**Boiling**	**Centrifugation**	**Quick-spin**	**Proteinase K**	**DNA extraction**	**Pre-PCR sample volume**	**PCR sample volume**	**Numbers of Pre-PCR replicates**	**Numbers of PCR replicates**	**Result**
**A. Establishment of *Borrelia* dPCR assay**															
I.	In sillico verification	Dry-lab verification of assay reactivity via Blast search	NA	NA	NA	NA	NA	NA	NA	NA	NA	NA	NA	NA	Described in this article
II.	Droplet count optimization	Effect of temperature on droplet counts	*Borrelia* gBlock	600 copies/PCR	PCR-grade water	NA	NA	NA	NA	NA	NA	5 µL	NA	Duplicates	Described in this article
III.	Annealing temperature	Assessment of the best annealing tempereature	*Borrelia* gBlock	600 copies/PCR	PCR-grade water	NA	NA	NA	NA	NA	NA	5 µL	NA	Duplicates	Figure 2A
IV.	Primer and probe concentration	Determining the optimal concentrations of reagents	*Borrelia* gBlock	600 copies/PCR	PCR-grade water	NA	NA	NA	NA	NA	NA	5 µL	NA	Triplicates	Figure 2B and C
V.	Combination of *Borrelia* and IPC assays	Assesment of compability	*Borrelia* and IPC gBlock	500 copies/PCR	PCR-grade water	NA	NA	NA	NA	NA	NA	5 µL	NA	Duplicates	Supplementary, Figure SA1
VI.	Frequency of false-positive droplets	Defines the number of positive droplets for a positive PCR result	*Borrelia* gBlock	0 copies/PCR	PCR-grade water, CSF matrix and Maxwell elution buffer	NA	NA	NA	NA	NA	NA	5 µL	NA	Octuplicates	Supplementary, Figure SA2
VII.	Assay reactivity (specificity and sensitivity)	Cross-reactivity and limit of detection	*Borrelia* gBlock, *Borrelia* species and genetic material from other microorganisms (Supplementary, Table S4)	0.125-128 copies/PCR	PCR-grade water	NA	NA	NA	NA	NA	NA	5 µL	NA	Duplicates and octuplicates	Figure 2D
**B. Optimization of "direct" pre-PCR method <u>without</u> DNA extraction**															
I.	Boiling (10 min at 95 °C followed by 10 min cooling at 4 °C)	Bacterial lysis	*B. garinii* Lu118	10-1000 cells per mL sample	CSF matrix	+	-	-	-	NA	1 mL	5 µL	Triplicates	Triplicates	Figure 3A
II.	Centrifugation (14,000 xg for 20 min.)	Concentration	*B. garinii* Lu118	10-1000 cells per mL sample	CSF matrix	+	+	-	-	NA	1 mL	5 µL	Triplicates	Triplicates	Figure 3B
III.	Quick-spin (10 sec. at 2000 xg)	Effect on rain	*B. garinii* Lu118	10-1000 cells per mL sample	CSF matrix	+	+	+	-	NA	1 mL	5 µL	Duplicates	Duplicates	Figure 3C
IV.	Proteinase K treatment (50 °C for 1 hour incubation)	Effect on lysis and rain	*B. garinii* Lu118	10-1000 cells per mL sample	CSF matrix	+	+	+	+	NA	1 mL	5 µL	Duplicates	Duplicates	Figure 3D
V.	Method sensitivity	Limit of detection	*B. garinii* Lu118	3-200 cells per mL sample	CSF matrix	+	+	+	-	NA	1 mL	5 µL	Triplicates	Duplicates	Figure 4
**C. Comparison of "direct" manual protocol to automated Maxwell DNA extraction method**															
I.	CSF matrix with and without leukocytes	PCR inhibition	*B. garinii* Lu118	50 cells per mL sample	CSF matrix	+	+	+	-	Maxwell	1 mL	5 µL	Triplicates	Duplicates	Figure 5
II.	Maxwell method sensitivity	Limit of detection	*B. garinii* Lu118	3-200 cells per mL sample	CSF matrix	-	+	+	-	Maxwell	1 mL	5 µL	Triplicates	Duplicates	Figure 6
**D. Test of patient samples with optimized pre-analytical method and dPCR assay**															
I:	Patients with and without definite LNB	Clinical evaluation	Patient samples	NA	CSF specimens	-	+	+	-	Maxwell	0.5-1 mL	5 µL	Single	Duplicates	Table 1 and Figure 7

dPCR = digital PCR
IPC = Internal positive control
NA = Not applicable
CSF = cerebrospinal fluid
+ = included in experimental setup
- = not included in experimental setup
